# Long-term dataset for contaminants in fish, mussels, and bird eggs from the Baltic Sea

**DOI:** 10.1038/s41597-024-03216-0

**Published:** 2024-04-20

**Authors:** Yosr Ammar, Suzanne Faxneld, Martin Sköld, Anne L. Soerensen

**Affiliations:** 1 Department of Environmental Monitoring and Research, Swedish Museum of Natural History, Stockholm, Sweden; 2 Department of Mathematics, Stockholm University, Stockholm, Sweden

**Keywords:** Environmental impact, Ocean sciences, Environmental monitoring

## Abstract

Widespread persistent contaminants are a global environmental problem. In the Baltic Sea, wildlife contamination was first noticed in the 1960s, prompting the Swedish Environmental Protection Agency to establish a comprehensive Swedish National **M**onitoring Programme for **Co**ntaminants in **M**arine Biota (MCoM) in 1978 run by the Swedish Museum of Natural History. Eight species have been analysed, four fish species (Atlantic herring, Atlantic cod, European perch, viviparous eelpout), one bivalve species (blue mussel), and egg from three bird species (common guillemot, common tern, Eurasian oystercatcher). Here, we present a dataset containing MCoM data from its start until 2021. It includes 36 sets of time-series, each analysed for more than 100 contaminants. The longest time-series is for common guillemot and starts in 1968. We describe the structure of MCoM including historic changes to the number of stations, sample treatment, analytical methods, instruments, and laboratories. The MCoM data is available at the Bolin Centre repository and on GitHub through our R package *mcomDb*. The latter will be updated yearly with new MCoM records.

## Background & Summary

The earth has a finite capacity to assimilate anthropogenically distributed contaminants including persistent organic contaminants and heavy metals^1^. Widespread release of contaminants is a global problem causing severe environmental and human health issues^2^. These contaminants contribute to altering ecosystems and the Earth´s system processes at different scales^3,4^. One example of such alteration is the Baltic Sea, a semi-enclosed coastal sea, where high concentrations of some organic contaminants and heavy metals have been a concern both historically and at present-day^5,6^.

In Sweden, a rapid increase in feather mercury concentrations of seed-eating birds from the 1940s was identified in studies covering the periods 1840 to 1960s^7–9^. This finding launched the start of an Environmental Specimen Bank in Sweden in the early 1960s (the world´s oldest). The Environmental Specimen Bank is located at the Swedish Museum of Natural History (SMNH). It saves, among other things, a collection of whole animals and tissues from marine, terrestrial and freshwater animals^10,11^.

**Fig. 1:**
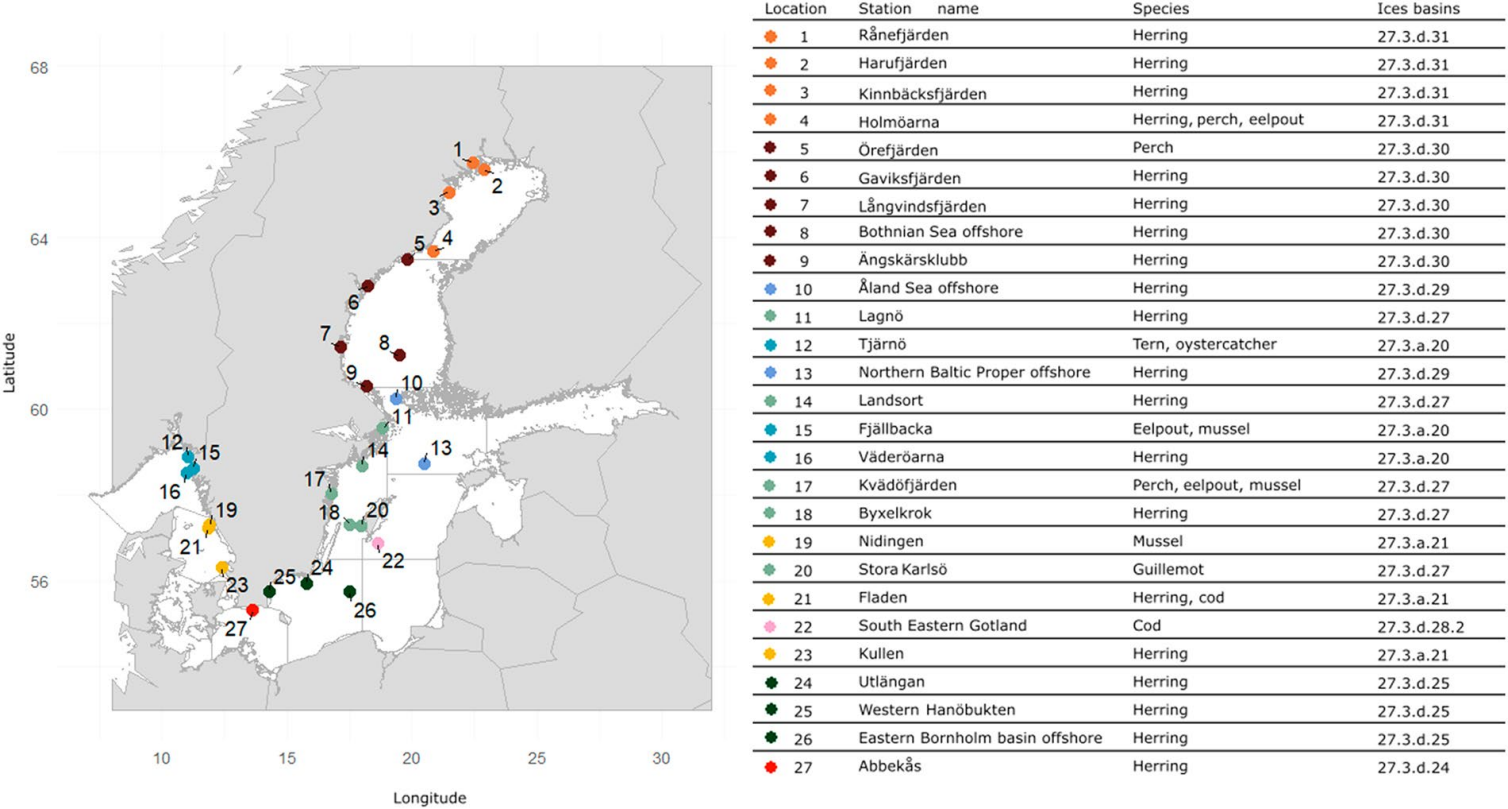
Stations within the MCoM. The colours indicate the ICES basin to which the station belongs (for info on ICES basin divisions see https://www.ices.dk/). Station names, species collected and ICES basin numbers are presented in the table to the right. A map with HELCOM basins is shown in Supplementary Information 2 (Figure S1).

Since the 1960s, a range of environmental threats have emerged in the Baltic Sea. For example, the impacts of high concentrations of organic contaminants such as polychlorinated biphenyls (PCBs), hexachlorocyclohexanes (HCHs), hexachlorobenzene (HCB), and dichlorodiphenyltrichloroethane (DDT) were observed in marine biota^12,13^. Although the latter contaminants were banned between the late 1970s and early 1980s, their levels did not immediately start to decline^14^. These findings instigated the modern biota contaminant monitoring in Sweden (Fig. 1).

In 1978, the Swedish Environmental Protection Agency (SEPA) established a comprehensive national contaminant monitoring programme for the environmental quality of Swedish wildlife^15–17^. The SMNH has been responsible for the parts of the programme focused on contaminants in marine, freshwater and terrestrial biota since then. Here, we present a comprehensive dataset with long-term contaminant observations from the Swedish National **M**onitoring Programme for **Co**ntaminants in **M**arine Biota (MCoM) including biological data of sampled specimens. Today, the programme includes 36 sets of time-series across 27 stations (at some stations multiple seasons or species are collected, Fig. 1) each factored with measurements of more than 100 contaminants. It covers eight species (Fig. 1): four fish species: Atlantic herring (*Clupea harengus*), Atlantic cod (*Gadus morhua*), European perch (*Perca fluviatilis*), viviparous eelpout (*Zoarces viviparus*); one bivalve species: blue mussel (*Mytilus edulis*); and egg from three bird species: common guillemot (*Uria aalge*), common tern (*Sterna hirundo*), and Eurasian oystercatcher (*Haematopus ostralegus*). These species were chosen to represent different trophic levels and ecosystem niches, with herring, which is found across the full geographical extent of the MCoM, being the cornerstone species of the programme. In the context of this work, “marine biota” refers to the fish, mussel and bird species that are part of the programme; we will cite the individual species as herring, cod, perch, eelpout, mussel, guillemot, tern and oystercatcher.

This programme was originally a tool for national contaminant monitoring purposes^18–21^, but has since 2008 also provided contaminant observations needed to live up to international obligations within the Marine Strategy Framework Directive^22^. Due to the long run time of the programme (>40 years) and the large number of contaminants (>100, Supplementary Table 1), numerous publications have used the data over the years. Overview reports were published sporadically in the first decades of the programme e.g.^20,21^, but in the past decade, a yearly report presenting temporal and geographical trends has been published (see^23^ for the latest report). Furthermore, in-depth reports and scientific publications have used smaller parts of the data. A few notable examples are Jörundsdóttir *et al*.^24^ and Miller *et al*.^25^ on organic pollutants in guillemot eggs, Nyberg *et al*.^14^ on non-dioxin like PCBs (ndl-PCBs), DDT, HCHs and HCB in biota, Faxneld *et al*.^26^ and Soerensen and Faxneld^19^ on Per- and Polyfluoroalkyl Substances (PFAS) in biota, Ek *et al*.^27^ on Polycyclic Aromatic Hydrocarbon (PAH) in mussels, and Soerensen *et al*.^28^ on selenium in herring. However, these publications only cover a fraction of the questions that can be explored with the MCoM data. For example, analyses have often been done with simple statistical tools (log-linear trend analysis or pair comparisons). The recent extension of methods for analyses of spatiotemporal variability focused on clustering and machine learning methods opens up a whole range of new scientific questions to be answered. Such inquiries could include questions about the factors driving spatiotemporal and seasonal variability in diverse contaminant groups, anticipating timeframe for regulatory measures to exert their influence, examining the impacts of climate and environmental changes on contaminant concentrations, and assessing how source appointments impact contaminant levels in the food-web. Furthermore, due to the yearly addition of new observations to the MCoM data, the information contained in the dataset is continuously extended, necessitating re-analysis of research questions previously investigated with the MCoM data.

Considering the potential of MCoM dataset for use within contaminant research for marine systems as well as transdisciplinary studies, our aim with this publication is to facilitate its accessibility. Systematic cleaning of the dataset was done during the transfer of old records from antiquated data formats to the new internationally accessible dataset format presented here. We provide background information on the analytical methods and instruments, as well as a description of how to use the data. The dataset is available in the Bolin Centre for Climate Research repository (Stockholm University) at 10.17043/ammar-2024-contaminant-monitoring-1^29^. In the future, the MCoM dataset will be updated yearly with new records and made publicly available in our package *mcomDb* (https://github.com/NRM-MOC/mcomDb) for the R statistical environment^30^.

## Methods

### Programme design

For a monitoring programme to be effective, it should have as few changes as possible over time in order to minimize noise in the data^31,32^. This requires sampling design and contaminants analyses that are consistent over time, with a focus on (1) collecting at the same stations, (2) collecting at the same time of year, (3) choosing specimens of the same age for the contaminant analyses, and (4) choosing laboratories for contaminant analyses that service the programme over many years if not decades. Still, when a programme runs as long as the MCoM, changes over time will happen. Below, we describe the essential structures that are the programme’s foundation and highlight the changes that have happened over time. These changes should be taken into consideration when analysing the data.

#### Stations

Sweden´s Exclusive Economic Zone (EEZ) extends along the Baltic Sea North-South gradient (Sweden´s east coast) to reach the North Sea (Sweden´s west coast). Currently, 36 sets of time-series across 27 sampling stations are part of the programme (Fig. 1). The number of stations has increased gradually (Supplementary Table 2), with the largest enlargement from 12 stations to 24 stations in 2007-2008^33^. Until then, the focus had been on coastal stations, but the first offshore stations were added in 2008. Collection sites unaffected by local sources were chosen for the programme and contaminant observations should reflect background concentrations in the Baltic Sea. Therefore, the concentrations can be used to look at regional signals in contaminant trends impacted mainly by diffuse sources. Furthermore, the established background level can be used as a reference for contaminated sites. Coordinates represent the general station location but the actual collection, in many cases done by local fishers, can deviate to some degrees from these coordinates. Similarly, bird eggs have been collected at nesting sites, which could deviate to some degrees from the station coordinates.

#### Collection season

When herring collection began at the stations Ängskärsklubb and Utlängan in 1972 it took place in the spring. However, since 1978, autumn collection has been the norm for both fish and mussels (with the exception of both spring and autumn time-series at Ängskärsklubb and Utlängan for herring). Autumn was chosen such that fish were collected outside the spawning season as discussed in Bignert *et al*.^21^. Bird eggs have been collected during the breeding season, which is in mid-April for the common tern and at the beginning and end of May for guillemot and oystercatcher, respectively. We associate the season in the dataset based on collection date with spring running from March to mid-July and autumn from mid-July to December. However, the majority of data is collected during a narrower range of months (Supplementary Information 2 Figure S2). Data from biota collected outside of these seasons were rare and have been excluded from the dataset.

#### Sampling design

Ten bird eggs of each species have been consistently analysed for contaminants every year either as individuals (guillemot eggs) or as homogenates (tern and oystercatcher eggs). Conversely, the sampling designs have evolved for fish and mussels; in the beginning of the MCoM, analyses of contaminants for individual specimens were prioritized for the majority of the substances. For instance, in the earliest years up to 20–25 individual herring specimens were analysed yearly at each station. To compensate for the increased costs of the geographical expansion and the addition of more contaminants into the MCoM over time, pooled samples (usually two pools of 10 or 12 individuals, larger pools for mussels), have been predominantly analysed since 2005 for each species at each station. However, individual samples are still analysed for herring from Ängskärsklubb, Landsort, Utlängan, Fladen, perch from Kvädöfjärden for metals, ndl-PCBs, DDTs, HCHs, HCB and Brominated Flame Retardants (BFRs). In addition to this, PFAS, dioxins and furans, and dioxin-like PCBs (dl-PCBs) have since 2018 been analysed individually from two herring stations every year (12 specimens) with different stations in a rotating scheme of 3-4 years. Not all herring sampling stations are used for this rotation as the small herring collected at the northern stations (stations 1–8, 10, 11, and 13 in Fig. 1) have too little tissue (muscle or liver) for individual analyses. The consequences of analysing pooled samples instead of individual samples were discussed in Bignert *et al*.^34^.

More specimens are usually collected each year than what is used for the programme. These specimens, and leftovers of the sampled specimens, are stored in the Environmental Specimen Bank at the SMNH.

### Contaminants

**Fig. 2:**
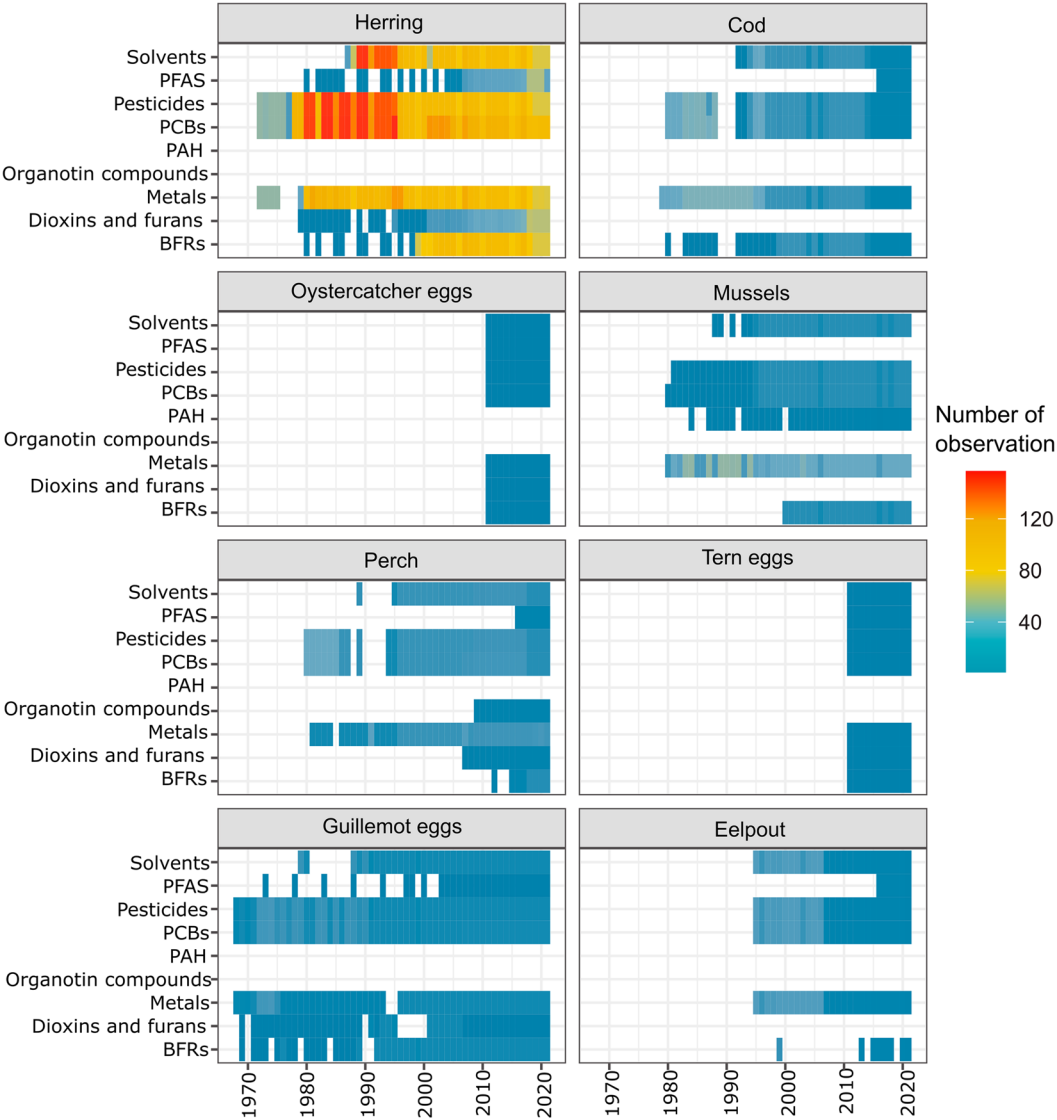
Number of individual or homogenate observations of contaminant concentrations for the eight substance groups per year for each species (as determined by unique “specimen_ID”; see Table 2). Retrospective analyses done for some substance groups are included. Number of individuals or homogenates observations of contaminant concentrations in the eight substance groups per year by station are shown in Supplementary Information 2 (Figure S3).

The MCoM dataset hosts 112 contaminants analysed over more than four decades for eight species. The contaminants include seven substance groups: metals, PFAS, PAH, dioxins and furans, PCBs, BFRs, solvents, organotin compounds, and pesticides (Fig. 2). For details on contaminants included in the substance groups see Supplementary Tables 1 & 2.

When the MCoM was first established, the focus was mainly on mercury, ndl-PCBs, DDT and HCB. Other substance groups and contaminants have since then been introduced. New substances are added as analytical methods develop and contaminants are acknowledged as being problematic through screening programmes. When new contaminants have been introduced to the programme, retrospective analyses have been conducted for selected sets of time-series (Fig. 2, Supplementary Table 2). This is the case for PFAS, PAH, dioxin and furans, and dl-PCBs and PBDEs.

Currently, emerging contaminants, such as modern pesticides and pharmaceuticals, are analysed through screening programmes in Sweden and the Baltic Sea, but are not yet included in the regular monitoring. This lag between new contaminants emerging and their inclusion in the regular monitoring can hamper the use of the data to provide an overall status assessment of the system.

### Sample preparation

Collection and sample preparations are performed according to the manuals for the collection, preparation and storage of biota at SMNH^35–37^. A short description follows below.

#### Protocol for fish

Adult individuals of a narrow length range are chosen by the fishers during the specimen collection based on knowledge of size-to-age correlation to minimize the within-year and between-year variation of the collected specimens. Due to changing ecosystem conditions (temperature, salinity, etc.) the size-to-age ratio changes across the geographical extent of the MCoM. The collected specimens are placed individually in polyethylene plastic bags, frozen and transported to the SMNH laboratory pending sample preparation. At the SMNH laboratory, the total body weight, total body length, sex, age, reproductive stage (herring), gonad weight (cod and perch), liver weight and sample weight are registered for individual specimens. The age is determined using scales for herring and otoliths for cod, perch, and eelpout. To minimize the variability that age difference can exert on concentrations, specifically on biomagnifying contaminants, fish sampled are chosen to represent a narrow age range (the choice is limited by the age of the collected specimen). The preferred ranges are: 3–5 years for herring, 3–4 years for cod from South-East Gotland, 2–4 years for cod from Fladen, 3–5 years for perch, and 3–6 years for eelpout. In addition to following these broader age ranges for the selection, care is taken to match the age of prior years at the specific stations.

Muscle and liver samples are used for the contaminant analysis of fish specimens. The sample tissue is selected based on the distribution of contaminants in the two tissues, and in some cases, to continue historic time-series for a specific tissue in the MCoM^38^. Muscle samples are taken from the middle dorsal muscle layer after the epidermis and subcutaneous fatty tissue have been carefully removed. At present, samples of 10–20 g muscle tissue are prepared for ndl-PCBs, DDTs, HCHs, HCB (ndl-PCBs, DDTs, HCHs, and HCB are referred to as organochlorines) and BFRs analyses, 15–25 g for dioxins and furans, and dl-PCB analysis and 1.5 g for mercury analysis. Due to the importance of cod liver oil, and the difficulty of detecting certain contaminants in its lean muscle, its liver is used in BFRs and organochlorines analyses. For the liver, samples of 0.5–1 g are prepared for metals other than mercury, 1 g for organotin compounds, and 0.5 g for PFAS analysis. More details on the collection and sample preparations for fish are found in^37^.

#### Protocol for blue mussels

All mussels from one station are placed together in a plastic bag, frozen and transported to the laboratory pending sample preparation as soon as possible. The specimen’s total shell length, shell and soft body weight are registered. Metals are analysed in individual mussels. Samples for organochlorines, BFRs and PAH analysis are prepared in pools of 20–75 specimens dependent on the available size of mussels at the individual stations. Differences in maximum size of mussels are determined by the salinity and other ecosystem variables resulting in those from the western stations close to the North Sea being larger than those further into the low salinity Baltic^36^. More information on the protocol for mussels can be found in^36^.

#### Protocol for bird eggs

Eggs are placed in an egg box and kept refrigerated until their preparation for the analyses. The length, width and total weight of bird eggs are recorded before the egg content is removed after taking away a small square of the eggshell. The eggs are collected soon after they are laid and hence an embryo should not exist. If embryo tissue exists in the egg, it is separated from the yolk and white before these are homogenized. The weight of the empty dried eggshell is then recorded and eggshell thickness is measured using a modified micrometre. Two grams of the homogenised egg content is prepared for mercury analysis, 2 g for the other metal analysis, 10 g for organochlorines and BFRs analyses, 30 g for dioxins, furans and dl-PCBs analysis and 1 g for PFAS analysis. More information on the protocol for bird eggs can be found in^35^.

### Laboratory analysis

Contaminant samples prepared at SMNH were sent to various laboratories that specialise in different contaminant groups. Table 1 presents an overview of the change of laboratories and the main analytical method for the studied substance groups over time; a more detailed description of specific protocols, methods and instruments by laboratory can be found in Supplementary Information 1 and in^18^.

**Table 1:** Overview of laboratories, methods and instruments used for contaminant analyses in biota over time.

Contaminant group	Laboratory	Analytical method	Instruments	Period
Metals	Isotope Technical Laboratory (ITL), the Royal Institute of Technology KTH, Sweden	ITL_Hg	Double-beam spectrophotometer	1972–1975
	Tekniska Röntgencentralen (TRC), the Royal Swedish Academy of Engineering Science in Stockholm, Sweden	TRC_Hg	Double-beam spectrophotometer	1968–1975
	Department of Environmental Assessment, Swedish University of Agricultural Sciences (SLU), Sweden	SLU_Hg	Double-beam spectrophotometer	1975–2006
		SLU_metals	AAS: Atomic Absorption spectrophotometer	1975–2003
			ICP-MS: Inductively coupled plasma mass spectrometer	2004–2006
	Department of Environmental Science, Stockholm University (SU), Sweden	SU_Hg	DMA80: direct mercury analyzer	Since 2007
		SU_metals	ICP-MS: Inductively coupled plasma mass spectrometer	
Polycyclic aromatic hydrocarbon (PAH)	Swedish Environmental Research Institute (IVL), Sweden	IVL_PAH	HPLC-FLC: high-performance liquid chromatograph and a fluorescence Detector	Since 2003
Organotin compounds (OT)	Swedish Environmental Research Institute (IVL), Sweden	IVL_OT	GC-MS-MS: gas chromatography connected to a triple quadrupole mass spectrometer	Since 2009
Dioxins and furans and dioxin-like PCBs	Department of Chemistry, Umeå University (UMU), Sweden	UMU_Dioxin_PCB	GC-HRMS: gas chromatography coupled to a high-resolution mass spectrometer	Since 1990 (and retrospective to 1969)
Organochlorines (ndl-PCBs, DDTs, HCHs, HCB) and BFRs	Department of Environmental Science, Stockholm University (SU), Sweden	SU_CLC_BFR	Packed-GC: Packed column gas chromatograph	1968–1987
			GC-ECD: gas chromatography connected to a mass spectrometer operating in electron capture detector	1988–2019
	Department of Chemistry, Swedish Food Agency (SFA), Sweden	SFA_CLC_BFR	GC-ECD: gas chromatography connected to a mass spectrometer operating in electron capture detector	Since 2020
PFAS	Department of Environmental Science, Stockholm University (SU), Sweden	SU_PFAS	LC-MS-MS: ultra-performance Liquid Chromatography coupled to a tandem mass spectrometer	2000–2019 (retrospectively to 1973)
	Department of Aquatic Sciences and Assessment, Swedish University of Agricultural Sciences (SLU), Sweden	SLU_PFAS	LC-MS-MS: ultra-performance Liquid Chromatography coupled to a tandem mass spectrometer	Since 2020
Stable isotopes	Stable Isotope Facility, UC Davis, California, USA	SI_UC_Davis	EA-IRMS: Elemental Analysis – Isotope Ratio Mass Spectrometer	Since 2013

The code for the analytical method corresponds to the code used in the database (Table 2). Detailed information on the analytical methods can be found in Supplementary Information 1.

Changes in laboratories and methods can result in the need to adjust/correct time-series if there is a systematic bias in measurements between laboratories or methods. When the laboratory analysing metals changed in 2007, an intercalibration analysis was conducted by Danielsson *et al*.^39^. While a significant difference between laboratories was found in the intercalibration experiment for some metals, we have subsequently not been able to replicate them by retrospective data analysis. Hence, we draw the conclusion that they were batch effects from the intercalibration samples rather than persistent differences between the laboratories, and recommend no correction to be done. Additionally, the Department of Environmental Science at Stockholm University (SU) performed the analyses of PFASs, organochlorines and BFRs until 2019. Since 2020, PFAS analyses have been carried out at the Department of Aquatic Sciences and Assessment at the Swedish University of Agricultural Sciences (SLU), whereas organochlorines and BFRs have been analysed by the Department of Chemistry at the Swedish Food Agency (SFA). An intercalibration study was conducted for these substance groups as presented in Faxneld *et al*.^40^. The study concluded that no conversions were needed to adjust the time-series because of the switch of laboratories, but that the limit of quantification (LOQ) was different for the two laboratories for PFAS. The datasets from these intercalibrations (intercalibration_metals.tsv, intercalibration_CLC.tsv, intercalibration_BFR.tsv, intercalibration_PFAS.tsv) are included in the repository for transparency. A further intercalibration study was conducted for the switch between the packed-GC and GC-ECD for organochlorines between 1987 and 1988 (Table 1). The intercalibration data is no longer available but Bignert *et al*.^20^ concluded that no corrections to the time-series were needed.

## Data Records

The MCoM dataset is available in tsv format (MCoM.tsv) in the Bolin Centre for Climate Research repository, accessible at 10.17043/ammar-2024-contaminant-monitoring-1^29^. It includes more than ten thousand individual specimens or homogenate observations (defined by unique “specimen_ID”; see Table 2), and more than two-hundred thousand records of unique contaminant concentrations (Fig. 2). The dataset comprises all contaminant records, station names, physical measurements, and laboratory information in a long format following the columns described in Table 2 (one table row for each measurement). In the repository, the intercalibration datasets described in Laboratory Analysis section (intercalibration_metals.tsv, intercalibration_CLC.tsv, intercalibration_BFR.tsv, intercalibration_PFAS.tsv) can also be found. In addition to the repository, all datasets is available in our R package *mcomDb*. MCoM dataset in the R package will continuously be updated in the years following the publication of this paper as new data becomes available. The R package includes a function that can shift the long table to a wide table format (*make_wide*). The latter format positions one row for each “specimen_ID” with observations for selected substance group contaminants in separate columns, which will make the dataset more accessible to some users. Documentation on the wide format can be found with the package. The two formats aim to accommodate the needs of different users.

**Table 2:** Description of database column as presented in the long format (MCoM.tsv) available in the Bolin Centre for Climate Research repository at 10.17043/ammar-2024-contaminant-monitoring-1^29^.

Database columns	Description
specimen_ID	Unique identifier for each specimen or homogenate
year	Sampling year presented in the format yyyy
date	Sampling date presented in the format yyyy-mm-dd
species	Full scientific name
species_EN	Common English name of the species
class	Species class: “Fish”, “Bird”, “Bivalve”
number_individuals	The number of specimens in the homogenate; else is 1.
sample_ID	Describes the sample sent to the laboratories for specific analysis and is a merger between the specimen_ID, the organ and the analytical method
substance_group	“Metals”, “PFAS”, “PCBs”, “Dioxins and furans”, “Pesticides”, “BFRs”, “Solvents”, “PAH”, “Organotin compounds”
contaminant	Name of contaminant as stated in Supplementary Table 1 (contaminant column)
value	Raw concentration reported by the laboratory, or threshold if value is below LOQ or LOD
unit	Unit for contaminant concentration
is_censored	TRUE or FALSE where TRUE refers to a value being an upper limit in the form of LOQ or LOD (See Supplementary Table 3)
LOQ_LOD	Indicates how the value relate to the method LOQ and/or LOD. A full description is found in Supplementary Table 3
uncertainty	The uncertainty level of the analysis. This is not calculated before 2011.
contaminant_full_name	Full name of the contaminant such as stated in Supplementary Table 3
contaminant_alt_name	Alternative short name of the contaminant widely used
CAS_number	The CAS or unique international identifier of the contaminant
suspected_outlier	TRUE or FALSE where TRUE refers to a suspected outlier based on median absolute deviation from linear model residual for the combination of station, species and contaminant.
laboratory	Different laboratories as described in Table 1 and Supplementary Information 1: “SU”, “UMU”, “SLU”, “IVL”, “SFA”, “ITL”, “TRC”
instrument	Instruments as described in Table 1 and Supplementary Information 1: “ICP-MS”, “DMA80”, “LC-MS-MS”, “GC-ECD”, “GC-HRMS”, “GC-MS”, “Packed-GC”, “Double-beam spectrophotometer”, “AAS” “HPLC-FLC”
analytical_method	Analytical methods as described in Table 1 and Supplementary Information 1:”SU_Metals”,”SU_Hg”,”SU_PFAS”,”SU_CLC_BFR”,”UMU_Dioxin_PCB”,”SLU_Hg”,”SLU_Metals”,”IVL_PAH”,”SFA_CLC_BFR”,”SLU_PFAS”, “IVL_OT”, “TRC_Hg”, “ITL_Hg”
sex	“Female”, “Male”, “Hermaphrodite”, “Immature”, “Mixed”, “Undetermined”, else is NA
total_length	Specimen total body length in millimetre (mm) for fish (including tail), mussel or egg, average if homogenate, else is NA
age	The specimen age, average if homogenate, else is NA
weight	Specimen total weight in gram (g), average if homogenate, else is NA
organ	Sampled organ for contaminant analysis: “Liver” or “Muscle” for fish, “Soft tissue” for mussels, and “Egg content” for birds.
fat_percentage	Fat percentage in the analysed organ specific to contaminant analysis.
dry_weight_percentage	Dry weight percentage of the analysed organ done by the laboratory with the contaminants analyses.
shell_thickness	Birds shell thickness in millimetres (mm), else is NA
d13C	Isotope ratio 13C/12C relative internal standards (δ^13^C; ‰)
d15N	Isotope ratio 15N/14N relative to air (δ^15^N; ‰)
C_dw_percentage	Total carbon in relation to dry weight
N_dw_percentage	Total nitrogen in relation to dry weight
station_name	Stations full name in English alphabet (ö changed to o and å,ä to a)
latitude	Latitude of the station (WGS84 projection system)
longitude	Longitude of the station (WGS84 projection system)
HELCOM_basin	HELCOM basin divisions to which belongs the station (see HELCOM basin divisions at https://helcom.fi/)
ICES_basin	ICES basin divisions to which belongs the station (see ICES basin divisions at http://www.ices.dk/)
ICES_station	Station code in ICES database
season	Sampling season “Spring” (March to mid-July) or “Autumn” (mid-July to December)

*Suspected outliers*: to help users identify suspected outliers we ran a linear model on log-transformed concentrations for each individual contaminant time-series (combination of contaminants, stations and species). We then identify outliers by the median absolute deviation (MAD) of model residuals

$$MAD=median\left(\left|{x}_{i}-median{\rm{(}}x{\rm{)}}\right|\right)$$

where, *x*_*i*_ is the value in the residual of the linear model and *median* (*x*) is the median value of the linear model’s residuals. An observation is then considered an outlier if its corresponding residual exceeds

$${T}_{\max }=median\,(x)+({\rm{\alpha }}\times \mathrm{MAD})$$

where α = 7.413. This choice of α corresponds to a cut-off range around the median equivalent to 5 times MAD. The value 7.413 is derived from 5 times the constant 1.4826, which is used to make the MAD consistent with the Normal distribution. The results are included as a column in the dataset (suspected_outlier in Table 2).

## Technical Validation

Here we present the technical validation that we conducted with a focus on improving user accessibility while enhancing the consistency and transparency of the MCoM data records. In addition to the quality checks and other technical validation done during this process, data validation has been performed continuously at SMNH and the contaminant analysis laboratories during the existence of the MCoM (see Supplementary Information 1).

The data collected through the MCoM is stored in-house at SMNH. Furthermore, every year new MCoM data are reported to a national data host who adds the data to an existing public record as well as reports it to the International Council for the Exploration of the Sea (ICES). Versions of the MCoM data are thus available (in Swedish only) at the current data host, the Geological Survey of Sweden (SGU; https://www.sgu.se/) and the international data host (ICES; https://www.ices.dk/). The Swedish data host and the reporting requirements from SMNH to the host have changed over the years. The latest update came in 2017, resulting in more complete records at the two external data hosts for data reported after this year. However, these changes have led to inconsistencies and incomplete historical records as detailed in Supplementary Table 4. Furthermore, the two external hosts are focused on storing data from multiple sources and therefore provide no description of the structure of the MCoM or its development over the years (including intercalibration datasets produced when laboratories changed), hampering the use of the data. Our work supplements this with a detailed description of the MCoM, its historical development, and intercalibration data.

We extracted historic data records stored in a deprecated data format in-house at SMNH and combined them with written records from the original sample collection and laboratory contaminant analysis results. This was done with the aim to create a publicly available dataset that standardizes the entire data record to the post-2017 reporting format, and extends the information to variables not currently found at the hosts. Updates of the published pre-2017 records found at the hosts include analytical methods and instruments, information on LOQ and LOD, and uncertainty of measurements. We further added information not currently included at the external hosts such as shell thickness for bird eggs, sample_ID and an identification of suspected outliers. For a detailed overview of pre and post 2017 reporting requirements and the not previously published additions to the MCoM database, see Supplementary Table 4.

**Fig. 3:**
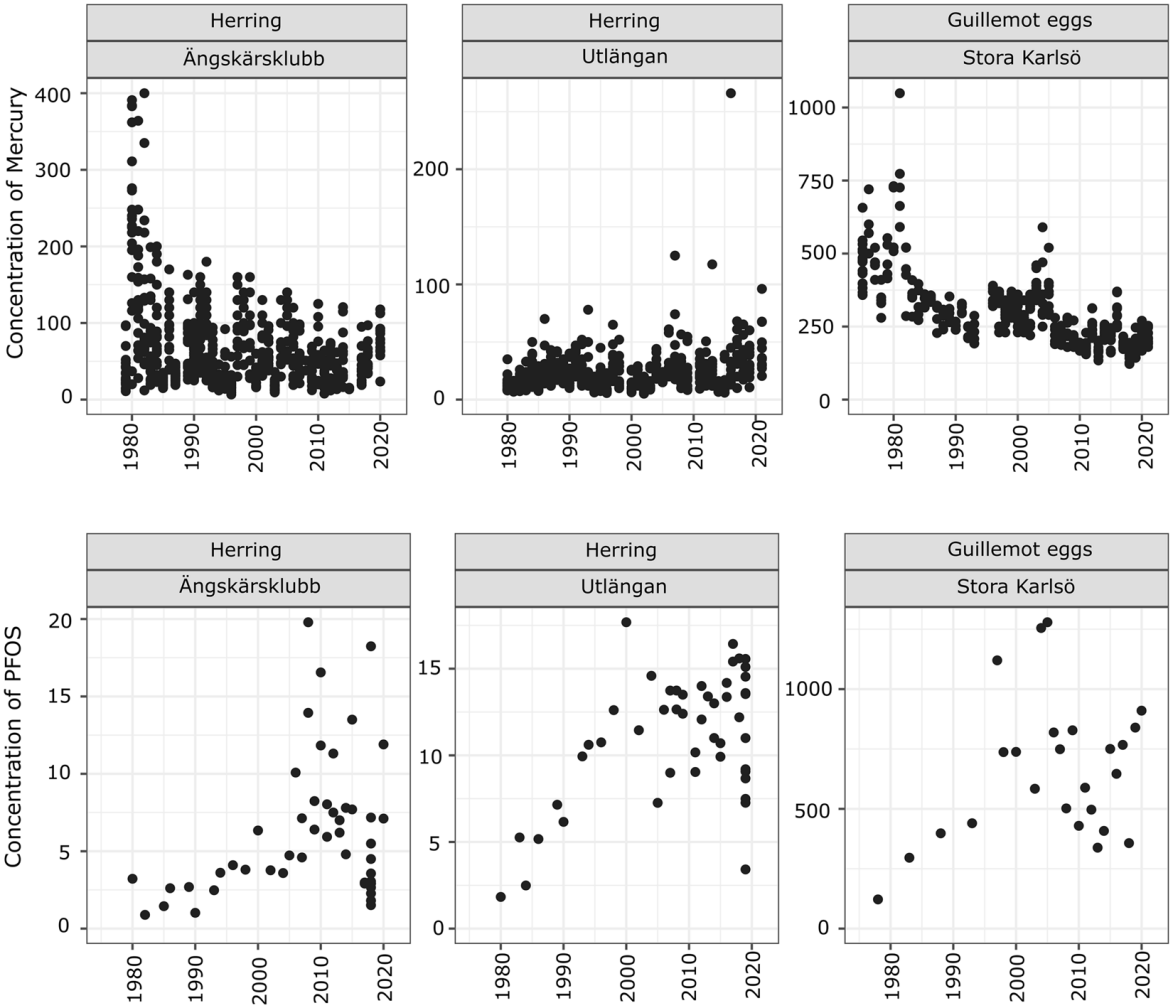
Time-series examples from the MCoM after technical validation for mercury (ng.g-1.ww-1) and PFOS (ng.g-1.ww-1) concentration in herring (Clupea harengus) from the autumn sampling at the stations: Ängskärsklubb and Utlängan and in the guillemot (Uria aalge) eggs from Stora Karlsö.

During the development of the new dataset, the data was thoroughly cleaned and quality controlled with an emphasis on the pre-2017 data. We conducted pattern tests of the contaminants time-series of all measurements to ensure they followed plausible and understandable patterns of variability over time, overall, by species, and by station. Figure 3 shows some typical time-series after validation. When encountering observations that did not adhere to the MCoM sampling design or were flagged as outliers, we went back to the original source of information (or the closest to this available) to check the validity of the observation and made corrections when errors were confirmed. This could for example be a check of the original paper recordings of incoming material or a closer look at information in the deprecated dataset or other electronically stored files related to the programme. Data from different laboratories analysing contaminants (sometimes reported in different units) were integrated and homogenized to the same unit. We saved the average values of the same tissue sample when duplicates were encountered (these could represent instances where uncertainty analysis had been conducted or where an original measurement had been questioned and a second analysis conducted). Inaccuracies in the fat percentages currently in the public records were further rectified by appropriately linking them to the correct contaminant concentration value, as reported by the laboratories.

We further standardized the seasons (spring and autumn) and removed observations from collections outside of these intervals. For all contaminants, we homogenized the acronyms and included information about the laboratories, analytical methods and instruments, in addition to the contaminant´s full name and corresponding CAS number if available. We unified the levels of factor variables such as sex to a common format. In addition, we verified the organs’ reporting, and translated variable names and other information stated in Swedish. With this technical validation, we have taken the necessary measures to ensure the reliability and integrity of the information within the developed dataset.

## Usage Notes

The dataset will be updated every year and data will be made available on GitHub in the R package *mcomDb:* https://github.com/NRM-MOC/mcomDb. The data descriptor was peer reviewed in 2024 based on the data available on the platform at the time.

### Supplementary information

Supplementary figures

Supplementary Tables

Analytical methods

## Data Availability

No code is available to reproduce the data. The R package *mcomDb* is publically available on GitHub (https://github.com/NRM-MOC/mcomDb) and includes a function for shifting the dataset from a long format to a wide format.
