# Supplementary material for: Long-term dataset for contaminants in fish, mussels, and bird eggs from the Baltic Sea
Source: Sci Data. 2024 Apr 20;11:400. doi: 10.1038/s41597-024-03216-0 (PMC11032401; doi:10.1038/s41597-024-03216-0)
Supplement: Supplementary file 1 — Supplementary figures [file 41597_2024_3216_MOESM1_ESM.pdf]

Long-term database for contaminants in fish, mussels, and bird eggs from the Baltic Sea

Authors
Yosr Ammar [1*], Suzanne Faxneld[1], Martin Sköld[1, 2], Anne L. Soerensen[1*]

Affiliations
1. Swedish Museum of Natural History, Department of environmental monitoring and research
2. Department of Mathematics, Stockholm University, Stockholm, Sweden

*Corresponding author(s): Yosr Ammar (yosr.ammar@nrm.se)
Anne L. Soerensen (anne.soerensen@nrm.se)

Supplementary Information 2: Supplementary figures

Below, we show a few supplementary figures referred to in the main text of the manuscript.

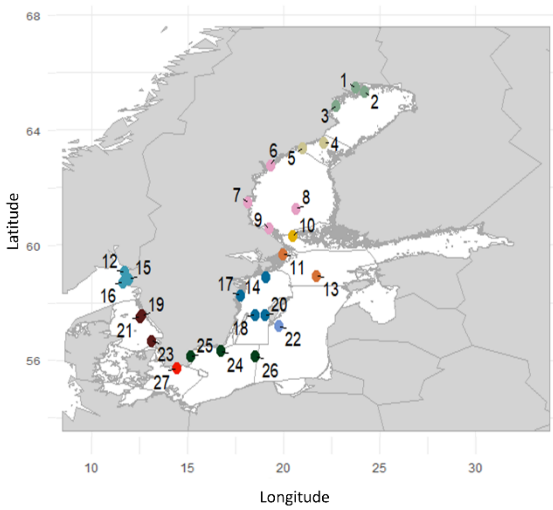

Location	Station name	Species	Helcom basins
1	Rånefjärden	Herring	Bothnian Bay
2	Harufjärden	Herring	Bothnian Bay
3	Kinnbäcksfjärden	Herring	Bothnian Bay
4	Holmöarna	Herring, perch, eelpout	The Quark
5	Örefjärden	Perch	Bothnian Sea
6	Gaviksfjärden	Herring	Bothnian Sea
7	Långvindsfjärden	Herring	Bothnian Sea
8	Bothnian Sea offshore	Herring	Bothnian Sea
9	Ängskärsklubb	Herring	Aland Sea
10	Åland Sea offshore	Herring	Aland Sea
11	Lagnö	Herring	Northern Baltic Proper
12	Tjärnö	Tern, oystercatcher	--
13	Northern Baltic Proper offshore	Herring	Northern Baltic Proper
14	Landsort	Herring	Western Gotland Basin
15	Fjällbacka	Eelpout, mussel	--
16	Väderöarna	Herring	--
17	Kvädöfjärden	Perch, eelpout, mussel	Western Gotland Basin
18	Byxelkrok	Herring	Western Gotland Basin
19	Nidingen	Mussel	Kattegat
20	Stora Karlsö	Guillemot	Western Gotland Basin
21	Fladen	Herring, cod	Kattegat
22	South Eastern Gotland	Cod	Eastern Gotland Basin
23	Kullen	Herring	Kattegat
24	Utlängan	Herring	Bornholm Basin
25	Western Hanöbukten	Herring	Bornholm Basin
26	Eastern Bornholm basin offshore	Herring	Bornholm Basin
27	Abbekås	Herring	Arkona Basin

Figure S 1: Stations within the MCoM. The colours indicate the HELCOM basin to which the station belongs (for info on HELCOM basin division see https://helcom.fi/). Station names, species collected and ices basin number are presented in the table to the right.

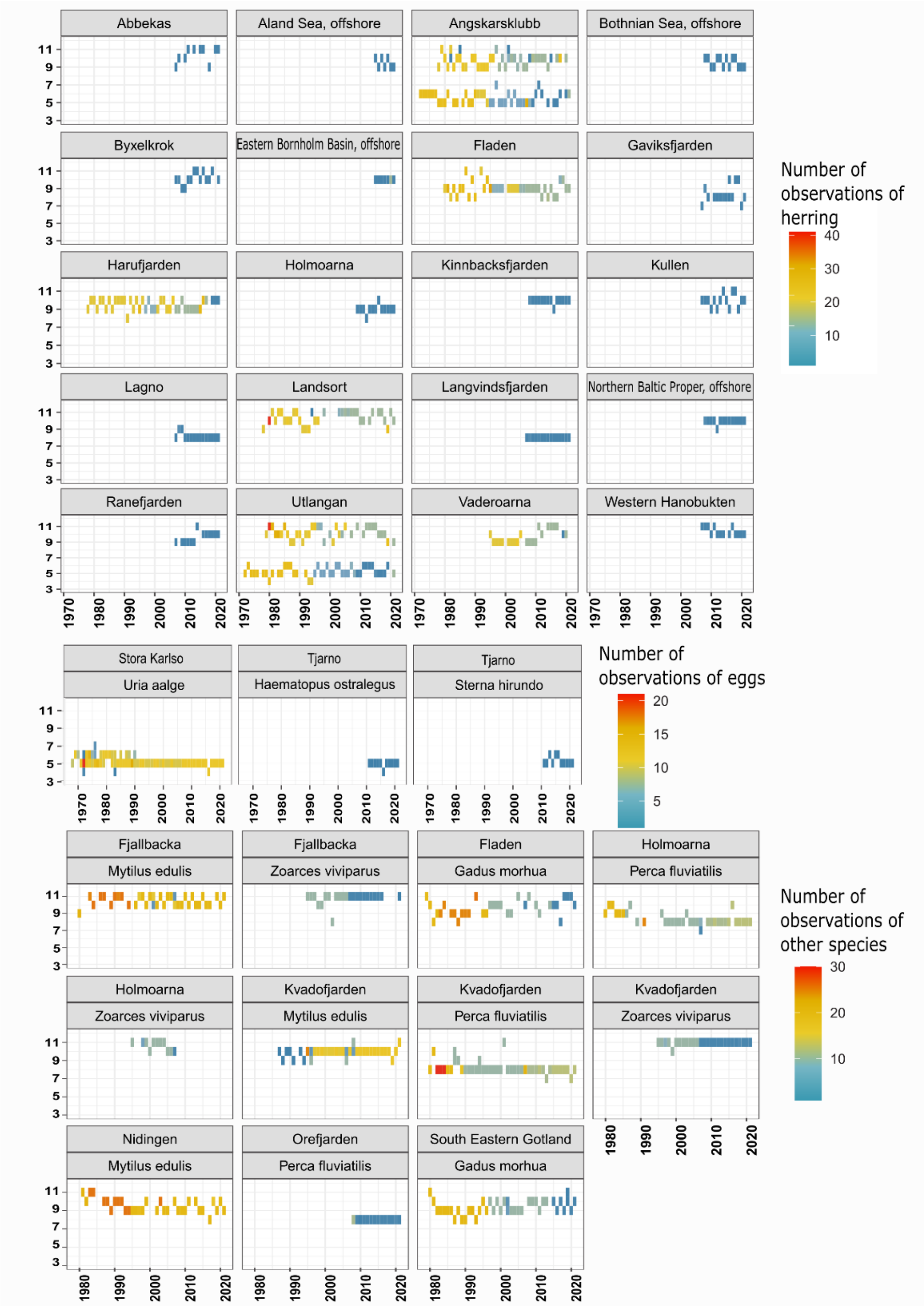

Figure S 2: Number of individuals or homogenates (as determined by unique "specimen_ID") by station and month over time and species in the programme. Spring collection season: March to mid-July, autumn collection season: mid-July to December.

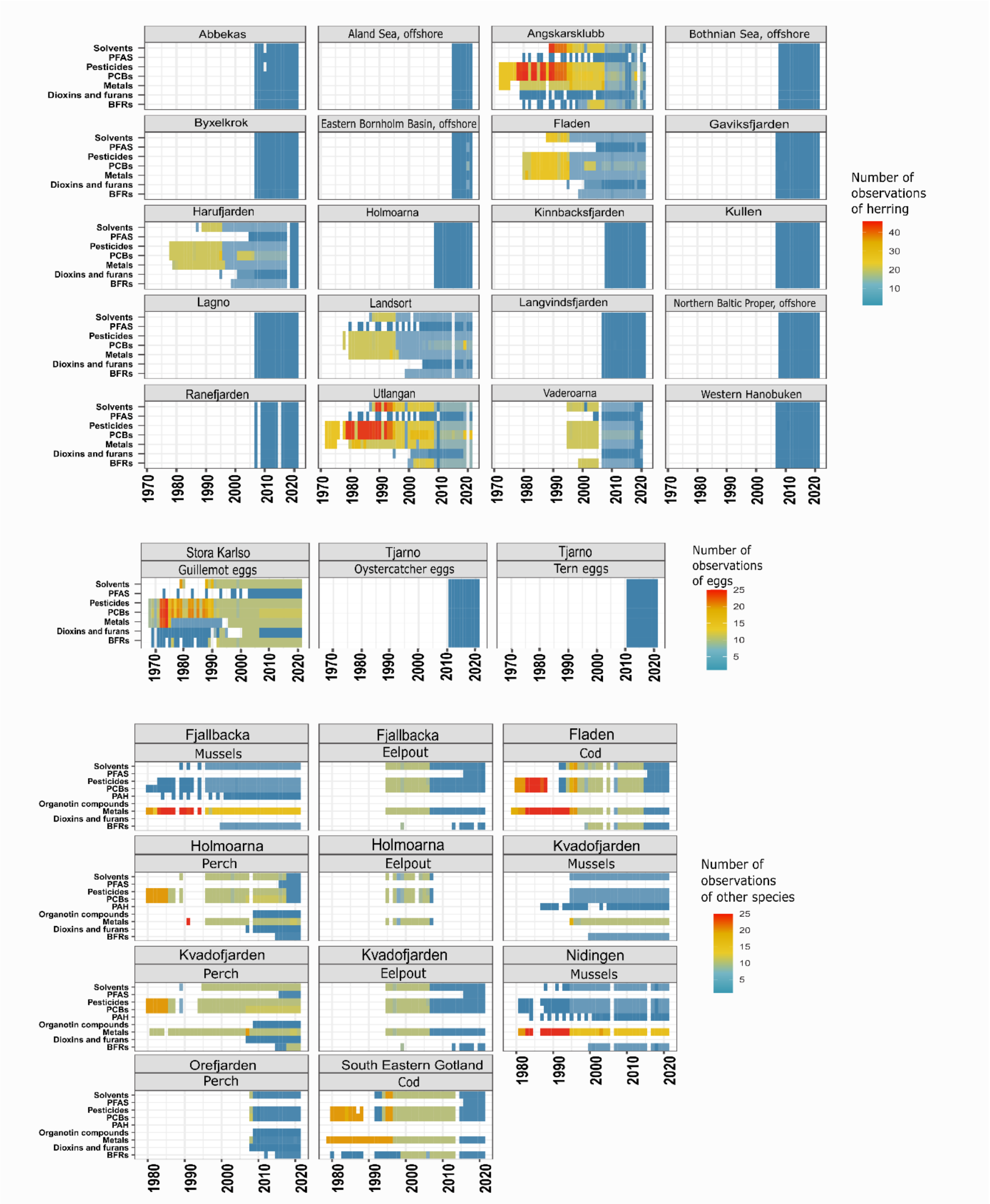

Figure S 3: Number of individuals or homogenates (as determined by unique "specimen_ID") observations of contaminant concentrations in the eight substance groups per year for each station and by species in the programme. Retrospective analyses done for some substance groups are included.
