# Supplementary material for: Long-term dataset for contaminants in fish, mussels, and bird eggs from the Baltic Sea
Source: Sci Data. 2024 Apr 20;11:400. doi: 10.1038/s41597-024-03216-0 (PMC11032401; doi:10.1038/s41597-024-03216-0)
Supplement: Supplementary file 3 — Analytical methods [file 41597_2024_3216_MOESM3_ESM.pdf]

Long-term database for contaminants in fish, mussels, and bird eggs from the Baltic Sea

Authors
Yosr Ammar [1*], Suzanne Faxneld[1], Martin Sköld[1, 2], Anne L. Soerensen[1*]

Affiliations
1. Swedish Museum of Natural History, Department of Environmental Monitoring and Research
2. Department of Mathematics, Stockholm University, Stockholm, Sweden

*Corresponding author(s): Yosr Ammar (yosr.ammar@nrm.se)
Anne L. Soerensen (anne.soerensen@nrm.se)

Supplementary Information 1: Analytical methods

Below, follows an in-depth description of the current and historic methods used for contaminants analyses within the Swedish National **M**onitoring Programme for **Co**ntaminants in **M**arine Biota (MCoM). The names indicated for each laboratory, analytical method and instrument (underlined acronyms) corresponds to the names found in Table 1 and in the database.

1. Metals

ITL_Hg* and *TRC_Hg*.** Before 1975, mercury in fish and mussels (ITL_HG***) was analysed at the Isotope Technical Laboratory (ITL), at the Royal Institute of Technology KTH. However, mercury in Guillemot eggs (***TRC_Hg***) was analysed at Tekniska Röntgencentralen (TRC) created by the Royal Swedish Academy of Engineering Science in Stockholm in 1937. In both laboratories, the quantification of mercury was done using a double-beam spectrophotometer. No more information is available about the laboratories.

***SLU_metals and SLU_Hg*.** Before 2007, metal analyses were performed at the Department of Environmental Assessment at the Swedish University of Agricultural Sciences (SLU) in Sweden. Digestion of freeze-dried fish liver and blue mussel soft tissue samples was performed in open vessels by the addition of HNO3 and incremental heating to 115-120 °C.

The quantification of metals (***SLU_metals***) was, before 2004, conducted on a Philips PU 9200 Atomic Absorption Spectrophotometer (AAS) with a graphite furnace and with a Philips PU 9390 electrothermal atomizer and autosampler (described by [1]). The quantification limit was estimated to be approximately 100 ng/g dry weight for zinc, approximately 10 ng/g dry weight for lead and copper, approximately 5 ng/g dry weight for cadmium and approximately 0.1 µg/g dry weight for nickel and chromium.

Between 2004 and 2006, metals in samples were quantified using inductively coupled plasma mass spectrometry (ICP-MS; Perkin Elmer ELAN 600). Certified reference material (CRM) used for metals except mercury were: DOLT-1: 1990-1991, DOLT-2: 1993-1997 and Bovine Liver B.L 1577b: 1997, TORT-2: 1997.

The analyses of mercury (***SLU_Hg***) were done according to May and Stoeppler [2], and Lindsted and Skare [3] using a double-beam spectrophotometer. The laboratory participated in the periodic Quality Assurance of Information on Marine Environmental Monitoring in Europe (QUASIMEME) intercalibration rounds. It has also participated in the programme for sampling quality control, QUASH CRMs used for mercury were DORM-2: 1994-1997.

SU_metals and SU_Hg. Since 2007, the Department of Environmental Science at Stockholm University (SU) in Sweden has determined metal concentrations in fish muscle (mercury) and liver (other metals than mercury), blue mussel soft tissue and bird eggs content.

The analytical methods for metals (***SU_metals***) in the liver were performed according to the Swedish standards SS-EN 13805:2009, *Foodstuffs – Determination of trace elements – Pressure digestion* (SS-EN 13805:2014 from 2018) and SS-EN ISO 17294-2:2005, *Water quality – Application of inductively coupled plasma mass spectrometry (ICP-MS) – Part 2: Determination of 62 elements* (SS-EN ISO 17294-2:2016 from 2018).

For mercury (***SU_Hg***), the analysis was conducted according to the US environmental protection agency method 7473, *mercury in solids and solutions by thermal decomposition, amalgamation and atomic absorption spectrophotometry,* using a direct mercury analyzer (DMA80).

The laboratory is accredited by SWEDAC (www.swedac.se) for the determination of dry matter (by freeze-drying), silver, aluminium, arsenic, cadmium, chromium, copper, nickel, lead, zinc and mercury in biota, and participates in the periodic QUASIMEME intercalibration rounds. CRMs used for mercury were DORM-2, DORM-3 and DORM-4 (dogfish muscle). For all other metals, the CRMs used were DOLT-3, DOLT-5 (dogfish liver), TORT-2 (lobster hepatopancreas), and NIST 1566 (oyster tissue).

2. Polycyclic aromatic hydrocarbon (PAH)

IVL_PAH. The analyses of PAH were carried out at the Swedish Environmental Research Institute (IVL). The extraction and analyses of the samples were performed according to the IVL-accredited method for PAH. The biota samples are spiked with recovery standard, homogenized in acetone and extracted in an ultrasonic bath. The extract is safeguarded and the samples are extracted once more with acetone and twice with pentane/ether. The extracts are combined and the organic compounds are extracted to an organic phase by liquid/liquid extraction with water and pentane, and further concentrated under nitrogen. The samples are hydrolyzed and pre-treatment procedures, such as fractionation of the organic compounds on silica are performed as additional "clean-up" procedures. Laboratory blanks follow the same procedures as samples in the analytical work.

The determination of PAH components was performed using a high-performance liquid chromatograph (HPLC, type Agilent 1290, with a 3 µm C18-column (Pursuit PAH 100* 3 mm, Agilent)). Before 2017, a 5 µm C18-column (Chromosphere PAH 100* 3 mm, Chrompack) was operated instead. A linear gradient elution programme was used, starting with acetonitrile/water 50:50 and ending with 100 % acetonitrile (Rathburne HPLC-grade). A fluorescence detector (FLC) Agilent 1260 (Varian Prostar 363 before 2017) with a wavelength programme optimized for each PAH was utilised for quantification. We refer to these two instruments as HPLC-FLC. The peak heights were registered with a chromatographic system from Varian (Star) before 2017 and from Thermo (Chromeleon 7.0) since 2017. The concentrations of 16 different PAH compounds were calculated by comparison to a certified standard, NIST, SRM 1647f (Priority Pollutant Polycyclic Aromatic Hydrocarbons in Acetonitrile).

All of the standards (internal and quantification standards) used were certified with known purity and precision. Since 2017, CRM was run in parallel to the mussel samples and used to check the method performance (NIST, SRM 2974a, Organics in freeze-dried mussels *Mytilus edulis* tissue).

3. Organotin compounds

IVL_OT. The analysis of organotin compounds has been carried out at IVL.

Before 2017, freeze drying samples was performed according to internal standards (monoheptyltin, diheptyltin) and 10 rehydrobromic acid (50 %). Since 2017, the internal standards changed (monoheptyltin, tripropyltin, tripropyltin and tetrapropyltin), and 10 mL hydrobromic acid (concentrated) were added. The mixture was extracted twice with 20 mL dichloromethane in a shaker. The organic extract was reduced in volume by evaporation under a stream of nitrogen and the solvent changed to 2 mL hexane: methanol 1:1. 40 µL 25 % sodium tetraethylborate in tetrahydrofuran was added and left to react for 2 hours at 90 °C before 2017 and at room temperature after 2017. After cooling, water was added and the hexane phase, together with an additional hexane extract, was reduced in volume and cleaned up on an alumina column with hexane as solvent.

A six-point calibration curve was prepared by diluting organotin mix 8 stock solution (LGC Promochem) to which fresh solutions of monophenyltin trichloride and diphenyltin dichloride in methanol were added. After ethylation using sodium tetraethylborate, water was added and the ethylates were extracted with hexane.

Instrumental analysis was achieved using a 7890A gas chromatograph connected to a 7004A triple quadrupole MS before 2017, which has changed since 2017 to a 7010 triple quadrupole mass spectrometer (GC-MS-MS) Agilent operated in electron ionization and multiple reaction monitoring (MRM) mode. For most compounds, two MRMs were recorded: one as a quantifier and the other as a qualifier to increase specificity. CRMs were used to check the performance of the method.

4. Dioxins, furans and dioxin-like Polychlorinated Biphenyls (PCBs)

UMU_Dioxin_PCB. The analyses of dioxins, furans and dioxin-like PCBs (dl-PCBs) were performed at the Department of Chemistry at Umeå University (UMU) in Sweden. The extraction method was described by Wiberg et al. [4], the clean-up method by Danielsson et al. [5], and the instrumental analysis using Gas Chromatography (GC) coupled to a high-resolution mass spectrometer (GC-HRMS) following Liljelind et al. [6]. The laboratory was participating in the annual FOOD intercalibration rounds, and was including laboratory CRM (salmon tissue) with each set of samples.

5. Organochlorines and brominated flame retardants (BFRs)

SU_CLC_BFR. The analyses of organochlorines and brominated flame-retardants (BFRs) were carried out at the Department of Environmental Science at SU in Sweden until 2019.

Before 1988, organochlorines were analysed by a packed column gas chromatography (Packed-GC) and the total sum of PCB was estimated from 14 peaks (peak 1-14) after calibration with Aroclor 1254 [7].

In 1988, the analysis on a capillary column was introduced allowing the analysis of individual congeners [8,9]. The extraction method originates from the method described by Jensen et al [7] where wet tissues were extracted with a mixture of polar and non-polar solvents. The PCBs that are included in the analysis are the non-dioxin like PCBs (ndl-PCBs) that are CB28, CB52, CB101, CB153, CB138, CB180 and the dl-PCB CB118. The organochlorines were analysed on a GC equipped with a µ-electron capture detector [8,9]. The BFRs were analysed by a GC connected to a mass spectrometer operating in electron capture detector negative ionization mode (NICI) [10]. We refer to the instrument as GC-ECD.

The quality control for organochlorines continuously improved and resulted in accreditation in 1999. The laboratory fulfilled the obligation of SS-EN ICO/IEC 17025:2005. The accreditation is valid for CB28, CB52, CB101, CB118, CB153, CB138, CB180, p, p'-DDT, p, p'-DDD and p, p'-DDE, HCB, α- Hexachlorocyclohexane (HCH), β-HCH, and γ-HCH in biological tissues. The BFRs were not accredited but the analysis of BDE28, BDE47, BDE99, BDE100, BDE153, BDE154 and Hexabromocyclododecane (HBCD) were in many ways performed with the same quality aspects as the organochlorines. The origin of all standards is well documented with known purity and certified concentration with uncertainty for the solutions.

To control impurities in solvents, equipment and glassware, one blank sample is extracted together with each batch of environmental samples. Co-elution of PCB congeners and pesticides in GC analysis is dependent on instrumental conditions such as column type, length, internal diameter, film thickness and oven temperature. To minimize possible co-elution, two 60 m columns were used in parallel, the commonly used 5 % diphenyl – 95 % dimethylpolysiloxane phase and the more polar 14% cyanopropylphenyl – 86 % dimethylpolysiloxane phase (before 2012, two 60 m columns were employed in parallel: the commonly used DB-5 and the more polar DB-1701). The only remaining known co-elution was for CB138, which co-elutes with CB163 [11]. Therefore, CB138+163 were reported together until 2019. Polybrominated diphenyl ether (PBDE) and HBCD were analysed on a 30 m column, 5 % diphenyl – 95 % dimethylarylenesiloxane phase (TG-5SilMS), monitoring m/z 79 and 81.

When introducing a new matrix one of the samples was re-extracted with a mixture of more polar solvents for control of no remaining contaminants in the matrix residual. Samples from new matrices/new sampling locations were also examined for suitable surrogate standards.

Three laboratory reference materials were used as extraction controls, chosen with respect to their lipid content and level of contaminants. Before 2014, herring and salmon were always utilized as reference materials for all contaminants. After 2014, the controls consisted of herring, pike and salmon muscle, homogenized in a household mixer and stored in aliquots in airtight bags of aluminium laminate at -80 °C. Since 1998, CRM 349, cod liver oil was also analysed twice a year for PCBs. In 2003, the laboratory changed to CRM 682 and 718 for mussel (soft tissue) and herring (muscle), respectively, being better representatives as they cover the whole extraction procedure. At each extraction event, one extraction control was extracted as well.

Proficiency testing. For PCBs and pesticides, the laboratory has participated in the periodic QUASIMEME proficiency testing since 1993, with two rounds every year, each one containing two samples. Around 95 % of all reported values were satisfactory according to QUASIMEME, meaning they were within ± 2 standard deviations of the assigned value. In 2000, the laboratory participated in the first interlaboratory study ever performed for PBDEs and HBCD, contaminants that were incorporated into the QUASIMEME proficiency-testing scheme since 2001. Around 85 % of the values that the laboratory has produced over the years were satisfactory according to QUASIMEME.

Detection limit. Calculation of uncertainty in measurement was based on the Nordtest Report TR 537 "Handbook for calculation of measurement uncertainty in environmental laboratories" [12], where the within-laboratory reproducibility was combined with an estimate of the method and laboratory bias. The within-laboratory reproducibility was calculated from laboratory reference material from more than 7000 PCB and pesticide values during a period of nearly 20 HCH proficiency testing of more than eight samples during at least 4 years. Only within-laboratory reproducibility was used for HBCD since no reliable proficiency testing (or CRM) exists today. Finally, the expanded uncertainty was calculated, using a coverage factor of 2 to reach approximately 95 % confidence level. The reproducibility of PCB and pesticide analyses

follows the theory stated by Horwitz et al. [13] where the relative standard deviation increase when the concentration level decreases. The reproducibility for the PBDEs and HBCD follows a function where the relative standard deviations increase first at the very lowest concentration. These values have been increasing with time, which means that, in recent years, more samples were used to calculate the uncertainty.

SFA_CLC_BFR. Since 2020, analyses of organochlorines and BFR were done by the Division for Laboratory Investigation and Analysis at the Swedish Food Agency (SFA). The laboratory is accredited according to SS-EN ICO/IEC 17025:2005 standard. The PCBs that are included in the analysis are the ndl-PCBs that are CB28, CB52, CB101, CB153, CB138, CB180 and the dl-PCB CB118. The analytical methods used for the analyses of PBDE, HBCD, PCB and chlorinated pesticides (HCB, α- HCH, β- HCH, γ- HCH, p, p'-DDT, p, p'-DDD and p, p'-DDE) have, with some modifications, previously been described in [14,15] and the extraction method originates from the method described by Jensen et al [7]. In brief, the samples have been extracted with acetone and hexane followed by a mixture of hexane and diethyl ether. The fat content was determined gravimetrically after evaporation of the organic solvents and then re-dissolved in n-hexane. The fat was removed from the extracts by sulphuric acid treatment. Further clean-up and fractionations were then made on a pre-washed silica gel column (4.5 g of 3 % deactivated silica gel).

For BFR, a first fraction was eluted with n-hexane and a second fraction containing the PBDE and HBCD was eluted with a mixture of dichloromethane/n-hexane (1:1 v/ v). The second fraction was reduced and transferred to an amber GC-vial. For PCB and chlorinated pesticides, a first fraction containing the PCB, HCB and p,p'-DDE was eluted with n-hexane and a second fraction containing the rest of the pesticides was eluted with n-hexane/diethyl ether (3:1 v/v). Both fractions were reduced and transferred to GC-vials. PBDE and HBCD have then been quantified with a GC/MS (Agilent Technologies 6890N/5975N) in electron capture negative ionization and selected ion monitoring mode (ECNI-SIM). 6 µl (2 x 3 µL) were injected in pulsed splitless mode using a programmable temperature vaporizing (PTV) injector with an initial temperature of 70 °C followed by rapid heating to 300 °C. The analytes were separated on a DB-5MS (15 m, 0.25 mm id, 0.1 µm film, JB&W Scientific) capillary column using a ramped carrier gas flow. The PCB and chlorinated pesticides were quantified on a GC (Agilent Technologies 6890N) equipped with dual capillary columns (Ultra-2; 50 m, 0.20 mm i.d., 0.33 µm film and DB-17; 60 m, 0.25 mm i.d., 0.25 µm film) and dual electron capture detectors (GC-ECD).

All solvents used were tested for trace amounts of analytes. The glassware was either rinsed with acetone or heated in an oven at 450 °C for at least three hours before use. Due to possible UV-induced degradation of the analytes, all sample extracts and standard solutions were stored in amber glassware. A chemical blank was included in each extraction series to monitor background levels. For each batch of samples, the corresponding blank sample levels of PBDE and HBCD were subtracted from the sample levels. A spiked in-house control sample was also included in each extraction series. The total relative mean recoveries are between 91% and 114% for BFRs, 71% and 113% for organochlorines. Precision expressed as relative standard deviation (RSD) is between 1 % and 21 %. The limit of quantification (LOQ) varied between 0.8 and 80 ng/kg fresh weight for BFRs and organochlorines, depending on the matrix and lipid content of the sample. The different analytes were identified by their retention times relative to the corresponding internal standards. The internal surrogate standards used for the quantification of BFRs is 13C-BDE-155 and for organochlorines PCB-112 and p,p'-DDD.

6. *Per- and Polyfluoroalkyl Substances* (PFAS)

SU_PFAS. Until 2019, the analyses of PFAS were performed at the Department of Environmental Science at SU. A sample aliquot of approximately 1.0 g homogenized tissue in a polypropylene (PP)-centrifuge tube was spiked with mass-labelled internal standards (18O- or 13C-labelled perfluoroalkyl sulfonates and carboxylic acids). Before 2016, 0.5 g of homogenized tissue was used for the bird eggs.

The samples were extracted twice with 5 mL of acetonitrile in an ultrasonic bath. Following centrifugation, the supernatant extract was removed and the combined acetonitrile phases were concentrated to 1 mL under a stream of nitrogen. The concentrated extract underwent dispersive clean-up on graphitized carbon and acetic acid.

A volume of 0.5 mL of the cleaned-up extract was added to 0.5 mL of aqueous ammonium acetate and volumetric standards M8PFOA and M8PFOS were added. Precipitation during overnight freezing occurred and the extract was centrifuged before the clear supernatant was transferred to an autoinjector vial for instrumental analysis.

Before 2010, aliquots of the final extracts were injected automatically on a high-performance liquid chromatography system (HPLC; Alliance 2695, Waters) coupled to an MS-MS (Quattro II, Micromass), which we refer to as LC-MS-MS. Compound separation was achieved on an Ace 3 C18 column (150 x 2.1 mm, 3 mm particles, Advanced Chromatography Technologies) with a binary gradient of ammonium acetate buffered methanol and water. The mass spectrometer was operated in negative electrospray ionization mode with the following optimized parameters: Capillary voltage, 2.5 kV; drying and nebuliser gas flow (N2), 300 and 20 L/h, respectively; desolvation and source temperature, 150 and 120 °C, respectively. The quantification was performed in selected reaction monitoring chromatograms using the internal standard method.

Between 2010-2019, aliquots of the final extracts were injected automatically on an ultra-performance liquid chromatography (UPLC) system (Acquity, Waters) coupled to a tandem mass spectrometer (MS-MS; Xevo TQS, Waters), which we refer to as LC-MS-MS. Compound separation was achieved on a BEH C18 UPLC column (1.7 µm particles, 50 × 2.1 mm, Waters) with a binary gradient of ammonium acetate buffered methanol in 2010 and 2011, and buffered acetonitrile afterwards and water. The mass spectrometer was operated in negative electrospray ionization, multiple reaction monitoring (MRM) mode. Quantification was performed by isotope dilution using exact matched (or structurally similar) isotope labelled internal standards.

The extraction method employed (except for the concentration step) has previously been validated for biological matrices and showed excellent analyte recoveries ranging between 90 and 110 % for PFCAs from C6 to C14 [16]. Recoveries for the analysis within MCoM were determined by spiked in-house fish control samples from 2018-2020. The recoveries ranged between 92 and 107% for all analytes except PFDS with an average recovery of 78%. The method for quantification limits (MQLs) for all analytes was determined either based on blank extraction experiments or if no blank contamination was detectable on the lowest calibration point and ranged between 0.036 and 0.088 ng/g wet weight for the different compounds.

SLU_PFAS. Since 2020, the analyses of PFAS were carried out at the Department of Aquatic Sciences and Assessment at SLU, Sweden. A sample aliquot of approximately 0.5 g homogenized tissue in a Precellys® Evolution vial was spiked with 5 ng each of a mixture of mass-labelled internal standards (^{13}C-labelled PFASs, $^{13}C_5$PFPeA, $^{13}C_5$PFHxA, $^{13}C_4$PFHpA, $^{13}C_8$PFOA, $^{13}C_9$PFNA, $^{13}C_6$PFDA, $^{13}C_7$PFUnDA, $^{13}C_2$PFDoDA, $^{13}C_2$PFTeDA, $^{13}C_3$PFBS, $^{13}C_3$PFHxS, $^{13}C_8$PFOS, $^{13}C_8$FOSA). The samples were homogenized and extracted twice with 3 mL acetonitrile in an ultrasonic bath followed by centrifugation. The supernatant extract was

transferred to a polypropylene centrifuge tube, and the combined acetonitrile phases were stored at -20 °C for approximately 12 hours. Thereafter, the supernatant was concentrated to 1 mL under a gentle steam of nitrogen. Then, graphitised carbon with acetic acid was added to the concentrated extract for clean-up. A volume of 0.5 mL of the treated extract was evaporated under a nitrogen stream until dryness and 1mL of methanol was added. Aliquots of the final extracts were injected into an HPLC system (ExionLC™, Sciex) coupled to an MS-MS (TripleQuad™3500, Sciex) that we refer to as LC-MS-MS. Compound separation was achieved on a Gemini C18 UPLC column (2 x 50 mm, 3 µm particles, Phenomenex) with a binary gradient of ammonium acetate buffered water and methanol. The mass spectrometer was operated in negative electrospray ionization mode.

Recoveries were determined by spiking a pool of fish samples collected within MCoM with a mixture of native standards (PFPeA, PFHxA, PFHpA, PFOA, PFNA, PFDA, PFUnDA, PFDoDA, PFTrDA, PFTeDA, L-PFBS, L-PFHxS, B-PFHxS, L-PFOS, B-PFOS, L-PFDS, FOSA) and the same mixture of mass-labelled internal standards as for the natural samples. The recoveries ranged from 68 to 145 %. The MQLs were determined based on either blank extraction experiments or, if no blank contamination was detectable, on the lowest calibration point. The MQLs ranged from 0.06 to 3.0 ng/g wet weight for the different PFASs. As quality assurance/quality control for the analysis, the laboratory annually analyses PFAS in a CRM fish muscle sample from QUASIMEME[17].

7. Isotopes

SI_UC_Davis. Material for stable isotope analysis was subsampled from the samples prepared for mercury analysis (fish muscle) or mercury and other metals analyses (mussel and egg) done at the Department of Environmental Science at SU. The analyses of stable isotopes were performed at the Stable Isotope Facility, UC Davis, California, USA.

Preparation of samples. Frozen samples were delivered from the SMNH to SU in acid-cleaned and pre-weighed plastic vials. The samples were freeze-dried for about one week and weighed before and after drying to check that the lyophilisation was successful and to enable the calculation of the dry matter (percentage). After the material had been secured for metal or mercury analysis, freeze-dried and pulverized tissue samples were transferred into 5x8 mm tin capsules (Säntis Analytical AG) and weighed. The target weight for dual-isotope analysis of biological samples was 1±0.2 mg. The filled tin capsules were formed into small spheres using clean metal tools and placed into 96-well microplates that were stored dry before being sent for analysis. Sample forms with sample type, weight and position in the microplate were sent electronically to the analysis laboratory. At least one blank sample (empty tin capsule) and one control sample (perch tissue) were included in each shipment of samples.

Analyses of C and N stable isotopes. Total carbon, total nitrogen and the isotope ratios 13C/12C and 15N/14N were determined by Elemental Analysis – Isotope Ratio Mass Spectrometry (EA-IRMS) using a PDZ Europa ANCA-GSL elemental analyser in tandem with a PDZ Europa 20-20 isotope ratio mass spectrometer. Laboratory standards that are calibrated to CRMs and of similar composition as the samples, as well as blanks, are repeatedly analysed within each sample run.

Delta values, $\delta^{13}C$ and $\delta^{15}N$, are reported in ‰ relative to international standards V-PDB (Vienna PeeDee Belemnite) for carbon and Air for nitrogen. Laboratory long-term standard deviation is 0.2 ‰ for $\delta^{13}C$ and 0.3 ‰ for $\delta^{15}N$.

References

1. Borg, H., Edin, A. & Sköld, E. Determination of metals in fish livers by flameless atomic absorption spectroscopy. *Water Res* (1981).
2. May, K. & Stoeppler, M. Pretreatment studies with biological and environmental materials. *Fresenius J Anal Chem* **317**, 248–251 (1984).
3. Lindsted, G. & Skare, I. Microdetermination of mercury in biological samples. *Analyst* **96**, 223–229 (1971).
4. Wiberg, K. *et al.* Enantioselective analysis of organochlorine pesticides in herring and seal from the Swedish marine environment. *Mar. Pollut. Bull.* **36**, 345–353 (1998).
5. Danielsson, C. *et al.* Trace analysis of polychlorinated dibenzo-p-dioxins, dibenzofurnas and WHO polychlorinated biphenyls in food using comprehensive two-dimensional gas chromatography with elctron-capture detection. *J. Chromatogr. Electrophor. Sep. Methods* **1086**, 61–70 (2005).
6. Liljelind, P. *et al.* Method for multiresidue determination of halogenated aromatics and PAHs in combustion-related samples. *Environ. Sci. Technol.* **37**, 3680–3686 (2003).
7. Jensen, S., Reutergårdh, L. & Jansson, B. Analytical methods for measuring organochlorines and methyl mercury by gas chromatography, edited. *FAO Fish. Tech. Pap.* **212**, 21–33 (1983).
8. Eriksson, U., Häggberg, L., Kärsrud, A. S., Litzén, K. & Asplund, L. *Analytical Method for Determination of Chlorinated Organic Contaminants in Biological Matrices*. 22 (1997).
9. Eriksson, U., Häggberg, L., Kärsrud, A. S., Litzén, K. & Asplund, L. *Analytical Method for Determination of Chlorinated Organic Contaminants in Biological Matrices*. 24 (2003).
10. Sellström, U., Kierkegaard, A., de Wit, C. & Jansson, B. Polybrominated diphenyl ethers and hexabromocyclododecane in sediment and fish from a Swedish River. *Environ. Toxicol. Chem.* **17**, 1065–1072 (1998).

11. Larsen, B. & Riego, J. Interference from 2,3,5,6-3′,4′-Hexachlorobiphenyl (CB 163) in the Determination of 2,3,4-2′,4′,5′-Hexachlorobiphenyl (CB 138) in Environmental and Technical Samples. *Int. J. Environ. Anal. Chem.* **40**, 59–68 (1990).
12. Magnusson, B., Näykki, T., Hovind, H., Krysell, M. & Sahlin, E. *Handbook for Calculation of Measurement Uncertainty in Environmental Laboratories,*. www.nordtest.info (2017).
13. Horwitz, W. & Albert, R. The Horwitz ratio (HorRat): A useful index of method performance with respect to precision. *J. AOAC Int.* **89**, 1095–1109 (2006).
14. Atuma, S. S. & Aune, M. Method for the Determination of PCB Congeners and Chlorinated Pesticides in Human Blood Serum. *Bull. Environ. Contam. Toxicol.* **62**, 8–15 (1999).
15. Lind, Y. *et al.* Polybrominated diphenyl ethers in breast milk from Uppsala County, Sweden. *Environ. Res.* **93**, 186–194 (2003).
16. Powley, C. R., George, S. W., Ryan, T. W. & Buck, R. C. Matrix Effect-Free Analytical Methods for Determination of Perfluorinated Carboxylic Acids in Environmental Matrixes. *Anal. Chem.* **77**, 6353–6358 (2005).
17. Proficiency testing WEPAL-QUASIMEME. *WUR* https://www.wur.nl/en/article/proficiency-testing-wepal-quasimeme.htm (2021).
